# Prolonged Pseudohypoxia Targets *Ambra1* mRNA to P-Bodies for Translational Repression

**DOI:** 10.1371/journal.pone.0129750

**Published:** 2015-06-18

**Authors:** Somayeh Pourpirali, Cristina Valacca, Paola Merlo, Salvatore Rizza, Silvia D’Amico, Francesco Cecconi

**Affiliations:** 1 Department of Biology, University of Rome Tor Vergata, Rome, Italy; 2 IRCCS Fondazione Santa Lucia, Rome, Italy; 3 Unit of Cell Stress and Survival, Danish Cancer Society Research Center, Copenhagen, Denmark; Universidade Federal do Rio de Janeiro, BRAZIL

## Abstract

Hypoxia has been associated with several pathological conditions ranging from stroke to cancer. This condition results in the activation of autophagy, a cyto-protective response involving the formation of double-membraned structures, the autophagosomes, in the cytoplasm. In this study, we investigated the cellular mechanisms regulating the autophagy gene *Ambra1*, after exposure to a hypoxia mimetic, cobalt chloride (CoCl_2_). We observed that, upon CoCl_2_ administration, activation of the apoptotic machinery was concomitant with down-regulation of the pro-autophagic factor Ambra1, without affecting transcription. Additionally, co-treating the cells with the caspase inhibitor z-VAD-FMK did not restore *Ambra1* protein levels, this implying the involvement of other regulatory mechanisms. Partial re-localization of *Ambra1* mRNA to non-translating fractions and cytoplasmic P-bodies was further detected. Thus, in this pseudohypoxic context, Ambra1 mRNA translocation to P-bodies and translational suppression correlated with increased cell death.

## Introduction

Multicellular organisms have developed oxygen-sensing systems to maintain oxygen homeostasis, which is essential for survival of the organism [1]. In fact, hypoxia has been detected in many pathological conditions such as coronary heart disease, stroke, and organ transplant injection [2]. Hif-1α (Hypoxia-inducible factor alpha) is a transcription factor that is rapidly activated in response to decreased levels of O_2_; this switches on, in turn, a subset of genes ensuring cell survival in hypoxic conditions [3]. Among the genes controlled by Hif-1α are VEGF (Vascular Endothelial Growth Factor) and EPO (Erythropoietin), both involved in erythropoiesis and angiogenesis to increase oxygen delivery to the hypoxic site [4,5]. Hypoxia also generally activates the cyto-protective response of autophagy in a Hif-1α dependent manner [3].

Autophagy is a highly conserved process of self-digestion through the lysosomal pathway. It involves the delivery of cytoplasmic components and organelles to the lysosomes through specific vesicles, termed autophagosomes [6]. Autophagy and the molecular components involved in this process have been widely studied. Ambra1 (Activating Molecule in Beclin 1-Regulated Autophagy) is a key molecule in autophagy upstream regulation and plays a role in neurodevelopment [7–9]. It encodes a 1,300 amino acid long protein that is highly conserved among vertebrates. Functional inactivation of the gene results in embryonic lethality due to severe neural tube defects [7].

Considering the involvement of defective autophagy in various pathological conditions, such as cancer and neurodegenerative disorders, several mechanisms regulating this process have been intensively investigated [10]. The most studied mode of regulation occurs through protein-protein interactions and post-translational modifications; However, new regulatory mechanisms are emerging, such as mRNA localization and processing through the miRNA machinery [11,12].

Once a stressor affects the cell, this should be able to respond rapidly by changing its proteome/transcriptome [13,14]. One mechanism suggested to this aim is mRNA localization. When one mRNA product is not required by the cell, it exits polysomes and assembles in an mRNP complex lacking translation initiation factor. This multi-protein RNA complex accumulates in processing bodies (P-bodies) to then proceed with decapping followed by degradation [15].

P-bodies are cytoplasmic structures composed of several core proteins (Edc3, decapping enzymes Dcp1/2, exonuclease Xrn1, etc.), some additional factors (such as GW182 and Ago1-4, etc.) and various mRNAs [16]. P-bodies also act as a storage site for those mRNAs that are not needed for a certain period of time. These silent intact mRNAs can exit P-bodies upon various stimuli to enter the translation machinery [15].

Cobalt Chloride (CoCl_2_) is a hypoxia mimetic which increases Reactive oxygen species (ROS) production and stabilizes HIF-1α protein through inhibition of prolyl hydroxylases [17], a process also known as *pseudohypoxia*. In this study we observed that prolonged exposure to CoCl_2_ resulted in a block of autophgy flux and induction of apoptosis, accompanied by gradual decrease of Ambra1 protein levels and re-localization of its transcripts to non-translating fractions and cytoplasmic P-bodies.

## Materials and Methods

### Chemicals and Antibodies

CoCl_2_ was purchased from Sigma-Aldrich. Caspase inhibitor z-VAD-FMK was obtained from BD Pharmingen. The following primary antibodies were used: rabbit anti-Hif1α (SCBT), rabbit anti-Ambra1 (SDI), rabbit anti-Actin (Sigma), rabbit anti-LC3 (Cell Signaling), rabbit anti-cleaved Caspase 3 (Cell Signaling), rabbit anti-p62 (MBL), rabbit anti-PARP. Goat anti-mouse and goat anti-rabbit HRP-conjugated secondary antibodies (Sigma) were used for western blotting. Alexa Fluor 488 and 555 conjugated secondary antibodies (Invitrogen) were used for immunofluorescence analysis.

### RNA extraction and reverse transcription

RNA was extracted from HeLa cells using QIAGEN mini RNeasy kit following the manufacturer’s instruction. Two μg of extracted RNA was used for reverse transcription and 500 ng of random primers for each μg of RNA was added to each tube. Samples were heated to 70°C for 5 min followed by immediate incubation on ice. M-MLV reaction buffer (1X), dNTPs, RNase inhibitor (1U/μl), and M-MLV reverse transcriptase (8U/reaction) (Promega) were added to the mix and were incubated for one hour at 37°C. Real-time PCR was performed using ABI lightcycler. 0.5 μg cDNA were used with 10 μl SYBR Green Master Mix (ABI), and 200 nM of each primer were added in a 20 μl reaction mix. The following primers where used for quantitative real-time PCR: Ambra1 (F: AACCCTCCACTGCGAGTTGA, R: TCTACCTGTTCCGTGGTTCTCC), hLC3B (F: CGGTGATAATAGAACGATACAAGG, R: CTGAGATTGGTGTGGAGACG), hTub-α6 (F: CCCCTTCAAGTTCTACTCATGC, R: ATTGCCAATCTGGACACCA), hL34 (F: GTCCCGAACCCCTGGTAATAGA, R: GGCCCTGCTGACATGTTTCTT), hp62 (F: AGCTGCCTTGTACCCACATC, R: CAGAGAAGCCCATGGACAG), and hNqo1 (F: CATCACAGGTAAACTGAAGGACC, R: TCAGCCACAATATCTGGGCTC).

### Cell cultures and Chemical Treatments

HeLa and HEK-293 cells were grown in Dulbecco’s modified Eagle’s medium with 10% fetal calf serum (FCS), 100 units/ml penicillin, 100 mg/ml streptomycin, and 2 mM glutamine, in a 5% CO_2_ atmosphere at 37°C. For CoCl_2_ treatment, cells were incubated with 500 μM CoCl_2_ from 0 to 24 hours. For z-VAD-FMK (10 μM) and MG132 (5 μM) treatments cells were incubated for the indicated time-points. For autophagy flux evaluation, chloroquine was added for the last hour of the CoCl_2_ treatment in the medium at a concentration of 20 μM. For Actinomycin D treatment, the drug was added for the last 4 hours of the CoCl_2_ treatment at a final concentration of 10 μM. For NAC treatment, this was added for the last 4 hours of CoCl_2_ treatment at a final concentration of 1 and 5 mM, respectively.

### ROS evaluation

To evaluate ROS relative concentration upon CoCl_2_ treatment, 30 min before the end of the experimental procedure, cells were incubated with 5μM DHE (Life Technologies) at 37°C. Cells were then washed twice in ice-cold PBS and collected. The fluorescence intensity of DHE, enhanced by the reaction with ROS, was analyzed by recording FL-2 fluorescence on a FACSVerse (BD Bioscences) flow cytometer.

### Protein extraction and Western blotting

Cells were lysed by addition of lysis buffer containing 50 mM Tris pH 7.5, 150 mM NaCl, 1% Triton, 1 mM sodium orthovanadate, 10 mM NaF and protease inhibitor cocktail (Sigma). The samples were incubated for 30 min on ice and centrifuged at 4°C for 10 min at 14,000 rpm. The supernatant containing the proteins was recovered and, after quantification by Bio-Rad protein quantification kit according to instructions, Sample Buffer 4X was added. Protein samples were separated by SDS-polyacrylamide gel (8% or 13.5% polyacrylamide, depending on the experiment) and transferred to nitrocellulose membrane. After 1 hour of incubation with 5% non-fat milk, membranes were incubated either over night at 4°C or 2 hours at room-temperature with anti- LC3 (1:1000), anti-Ambra1 (1:2000), anti-HIF1-α (1:1000), anti-p62 (1:1000), anti-actin (1:1000), anti-caspase 3 (1:300), and anti-PARP (1:1000) primary antibodies. After extensive washing, the membranes were incubated for 1 hour at room temperature with secondary antibodies and immunoreatcive bands were visualized using an enhanced chemiluminescence reagent (Millipore). Acquisition was performed with Fluorchem SP (Alpha Innotech).

### RNA-FISH

QuantiGene View RNA ISH cell assay kit was obtained from Affymetrix. The experiment was conducted as instructed. In brief, HeLa cells were grown on poly-lysine coated glass slides, 96-well plates, or 384-well plates to 70% confluence. Cells were washed with PBS and fixed in 4% formaldehyde for 30 min. After washing with PBS 1X, they were permeablized with Detergent Solution for 5 min followed by protease digestion (1:8000) for 10 min. Working Probe Sets were added and the slides were incubated at 40°C for 3 hours. Consequently, they were incubated with pre-Amplifier, Amplifier, and Label Probe Mix, each step for 30 min at 40°C. Finally, the slides were counterstained with DAPI and the images were acquired by using a confocal microscope (see below). RNA probes were designed by Affymetrix against human Ambra1 and human Beclin 1.

### Immunocytochemistry

Cells were washed with PBS 1X and fixed with 4% paraformaldehyde for 15 min at room temperature. After several washes with PBS they were permeabilized with 0.5% Triton X-100 in PBS for 5 min before immunostaining. After incubation with 5% BSA for one hour at room temperature, the cells were incubated over night with anti-Ge-1 (1:400) primary antibodies. Detection of the primary antibodies was performed using 1:400 Alexa Fluor 488 goat anti-rabbit IgG and Alexa Fluor 555 goat anti-mouse IgG secondary antibodies for 1 hour at room temperature. For nuclei detection, cells were counterstained with DAPI.

Confocal images were acquired with Confocal laser scanning microscope Olympus FV 1000 using laser 405 nm (diode) for blue channel (DAPI), 488nm (argon) for green channel (FITC) and 543nm (HeNe) for red channel (TRITC). Oil objective 60x (NA 1,35) with optical zoom 3x were used. Fluorochrome unmixing was performed by automated-sequential collection of multi-channel images to reduce spectral cross-talk between channels. Ambra dots colocalization with GE-1 has been done using ImageJ software; For control and pseudohypoxia conditions 26 (5 fields) and 20 cells (4 fields) have been counted, respectively. For apoptotic nuclei analysis, DAPI was added in the medium of living cells; after 10 minutes of incubation, 10 random images per condition were taken and apoptotic and normal nuclei were counted.

### Linear sucrose gradient fractionation

Polysome profile analysis was performed using linear sucrose gradient fractionation (LGS). First, cells were incubated with 100 μg/ml cycloheximide (Sigma) for 15 minutes at 37°C. Then, they were washed with PBS containing 100 μg/ml cycloheximide and lysed with 500 μl LGS buffer (100 mM KCl, 20 mM Tris, pH 7.5, 5 mM MgCl_2_, 0.4% NP-40, 100 μg/ml cycloheximide, 0.1 U RNase inhibitor (Promega) and complete EDTA-free protease inhibitor). Cell lysate were centrifuged at 14,000 rpm for 15 min at 4°C and supernatant was recovered. The supernatant was loaded onto 15–50% sucrose gradient. Ultra-centrifugation was performed for 110 min at 37,000 rpm at 4°C in a Beckman SW41 rotor. Each gradient was collected as 10 fractions (1 ml each) in a tube containing SDS with the final concentration of 1% and RNA-spike (20 pg/fraction). Fractions were monitored by continuous OD_254_ measurement; 100 μg/ml Proteinase K was added to the samples followed by 1 hour incubation at 37°C. Total RNA was extracted from each fraction using phenol-chloroform and isopropanol precipitation.

## Results

**Fig 1:**
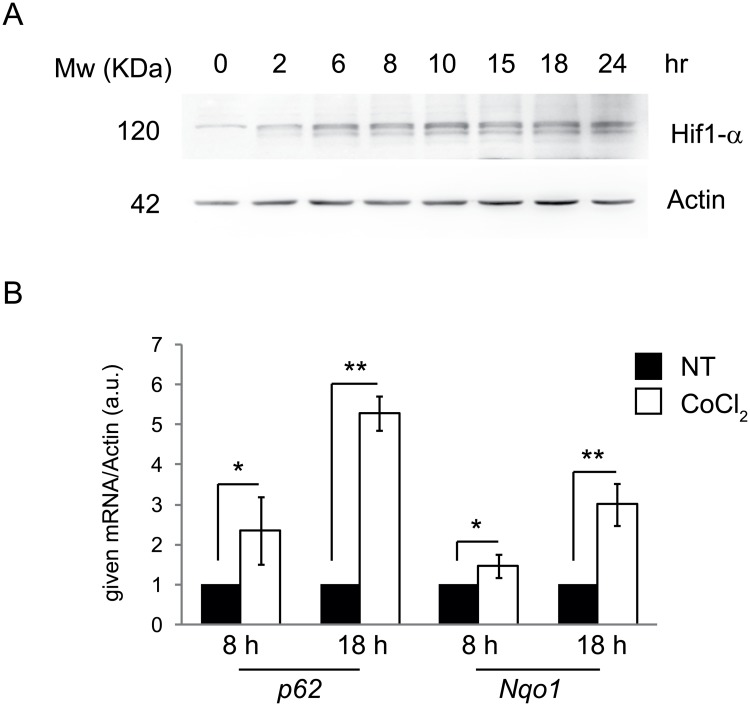
CoCl_2_ induces pseudohypoxia. HeLa cells were treated with 500 μM CoCl_2_; The induction of pseudohypoxia were detected by Hif1-α protein expression at 0, 2, 6, 8, 10, 15, 18, and 24 hr of treatment (A) and by *p62* and *Nqo1* mRNA expression at 8 and 18 hr of treatment (B). P-values <0.05 (*) and <0.005 (**) are considered statistically significant.

**Fig 2:**
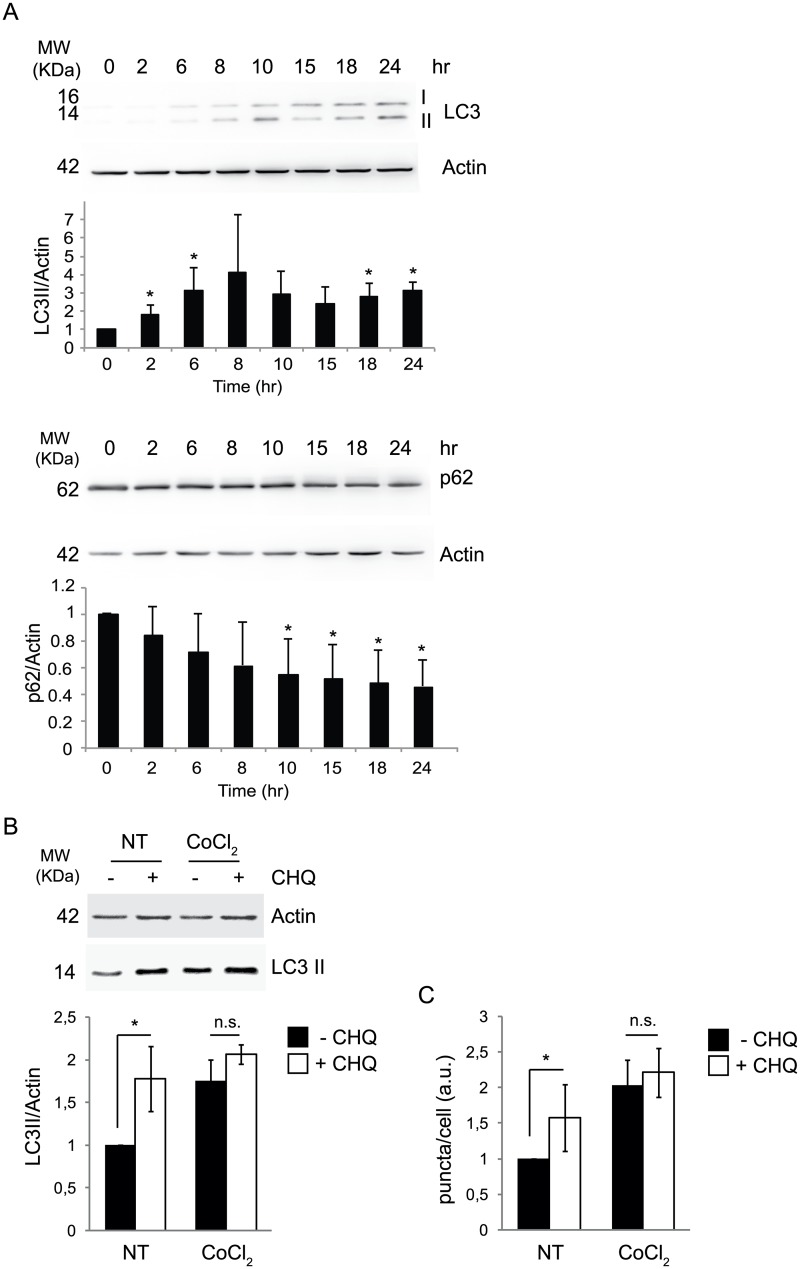
Persistent pseudohypoxia results in a block of autophagy flux. HeLa cells were treated with 500 μM CoCl_2_ and autophagy (indicated by p62 and LC3II/Actin ratio) were analysed at 0, 2, 6, 8, 10, 15, 18, and 24 hr (A). Autophagy flux was monitored by LC3 II accumulation after adding chloroquine (CHQ) at a final concentration of 20 μM for the last 1 hr of treatment in presence of CoCl_2_ or not (NT) (8 hr total treatment) by W.B. (B) and IF (C). The graphs represent the densitometric analysis of three different experiments ± SD. *: P-value < 0.05 is considered statistically significant, n.s. = not significant.

**Fig 3:**
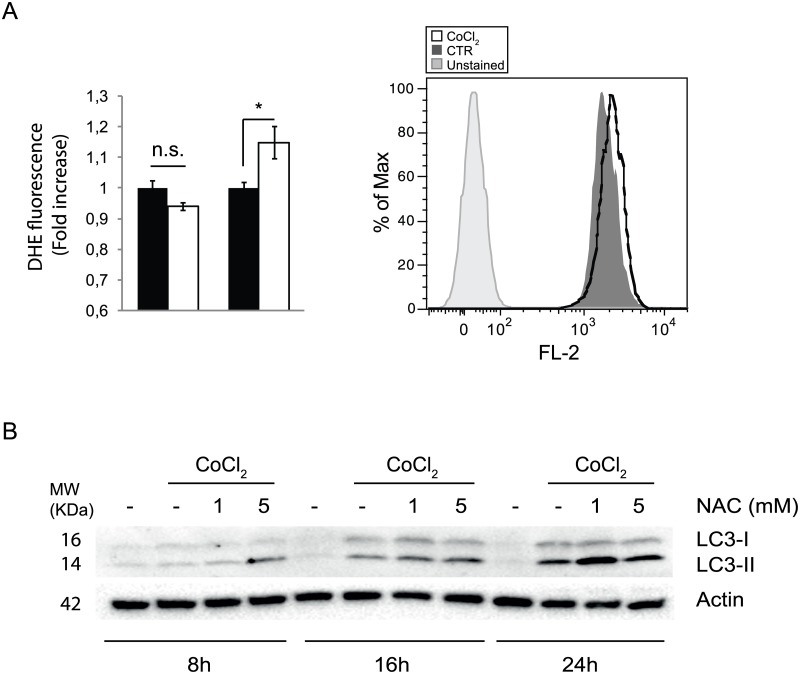
ROS production is not a direct consequence of CoCl_2_ treatment. The presence of ROS was evaluated by FACS analysis in HeLa cells treated with 500 uM CoCl_2_ for 8 and 16 hr (A). Accumulation of LC3 II after 500 uM CoCl_2_ treatment for 8, 6 and 24 hours was monitored by W.B. adding the ROS scavenger NAC for the last 4 hours of treatment at a final concentration of 1 and 5 mM (B). The graphs represent the densitometric analysis of three different experiments ± SD. *: P-value < 0.05 is considered statistically significant, n.s. = not significant.

To study the regulatory mechanisms of hypoxia-induced autophagy, HeLa cells were treated with 500μM CoCl_2_ for 24 hours, a condition termed ‘pseudohypoxia’. Induction of pseudohypoxia was confirmed by detecting in a time-course Hif1-α protein levels (Fig 1A), which were increased as early as 2-6h after treatment with CoCl_2_. To further verify hypoxia induction we performed a Real-Time PCR on *p62* and *Nqo1* mRNAs, two targets of the transcription factor Nfr2, known to be activated during hypoxia; Indeed, both after 8 and 18 hours of CoCl_2_ treatment, the two mRNAs are significantly upregulated (Fig 1B). Autophagy was then monitored by western blot analysis of two common markers, p62 and LC3. As shown in Fig 2A, p62 protein levels decreased with time, accompanied by more conversion of LC3I to LC3II. In order to understand whether the accumulation of LC3II was due to an activation of autophagy or rather to a block of this process, which would impair its degradation, we analyzed the autophagy on-rate/off-rate (autophagy flux) by using chloroquine, an inhibitor of the autophagosome/lysosome fusion. We found that 8 hours after treatment with CoCl_2_ there is no production of new autophagosomes; instead, the autophagy flux is blocked, leading to LC3II accumulation (Fig 2B). To further corroborate this data, we next analysed LC3 dots by immunofluoresce in cells treated with CoCl_2_, both in basal and autophagy-blocked conditions; as shown in Fig 2C, CoCl_2_ inhibits LC3 dots formation after chloroquine, this confirming a block of the autophagy flux (Fig 2C and S1 Fig). Of note, an expected ROS increase due to pseudohypoxic conditions, is induced only 16 hours after CoCl_2_ administration, this indicating that ROS are not responsible of the observed effects on autophagy, whose block is detectable as soon as 8 hours after treatment (Fig 3A). In agreement with this conclusion, treatement with the ROS scavenger NAC has modest if any effects on LC3II accumulation (Fig 3B).

**Fig 4:**
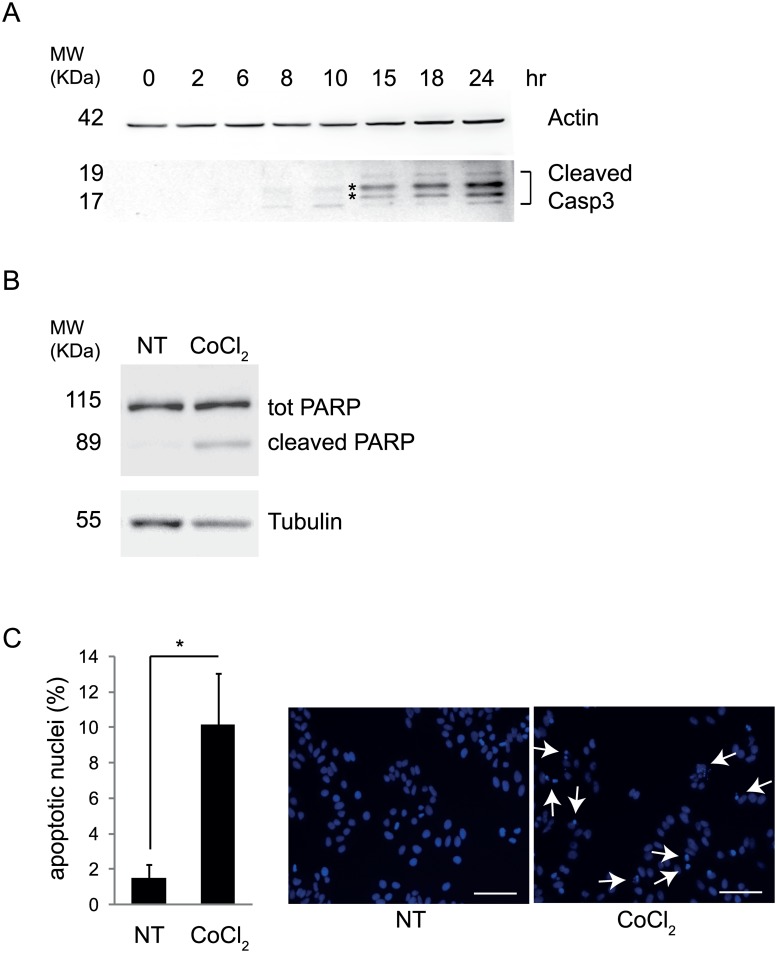
Hypoxia mimetic CoCl_2_, induces apoptosis. HeLa cells were treated with 500 μM CoCl_2_. Apoptosis was analyzed by monitoring cleaved Caspase3 at 0, 2, 6, 8, 10, 15, 18, and 24 hr of treatment (A), cleaved PARP at 24 hr (B) and by counting apoptotic nuclei stained with DAPI at 24 hr of treatment (C);. Asterisks point to aspecific bands. Experiments have been repeated three times with similar results. Representative images of nuclei stained with DAPI are shown (scale bar 100 μm). The graph represents the densitometric analysis of three different experiments ± SD. *: P-value < 0.05 is considered statistically significant.

Since several studies show the activation of apoptosis following CoCl_2_ treatment [18], this process was also analyzed in treated cells. As shown in Fig 4A, at later time points, cleavage of caspase 3 (Casp3) indicates apoptosis induction. As a consequence of caspase activation, after 24 hours of CoCl_2_ treatment, we observe cleavage of PARP [Poly (ADP-ribose) polymerase], a hallmark of apoptosis (Fig 4B). In line with this finding, a count of apoptotic nuclei revealed an increased cell death following the treatement, confirming that, indeed, CoCl_2_ treatment leads to apoptosis (Fig 4C).

**Fig 5:**
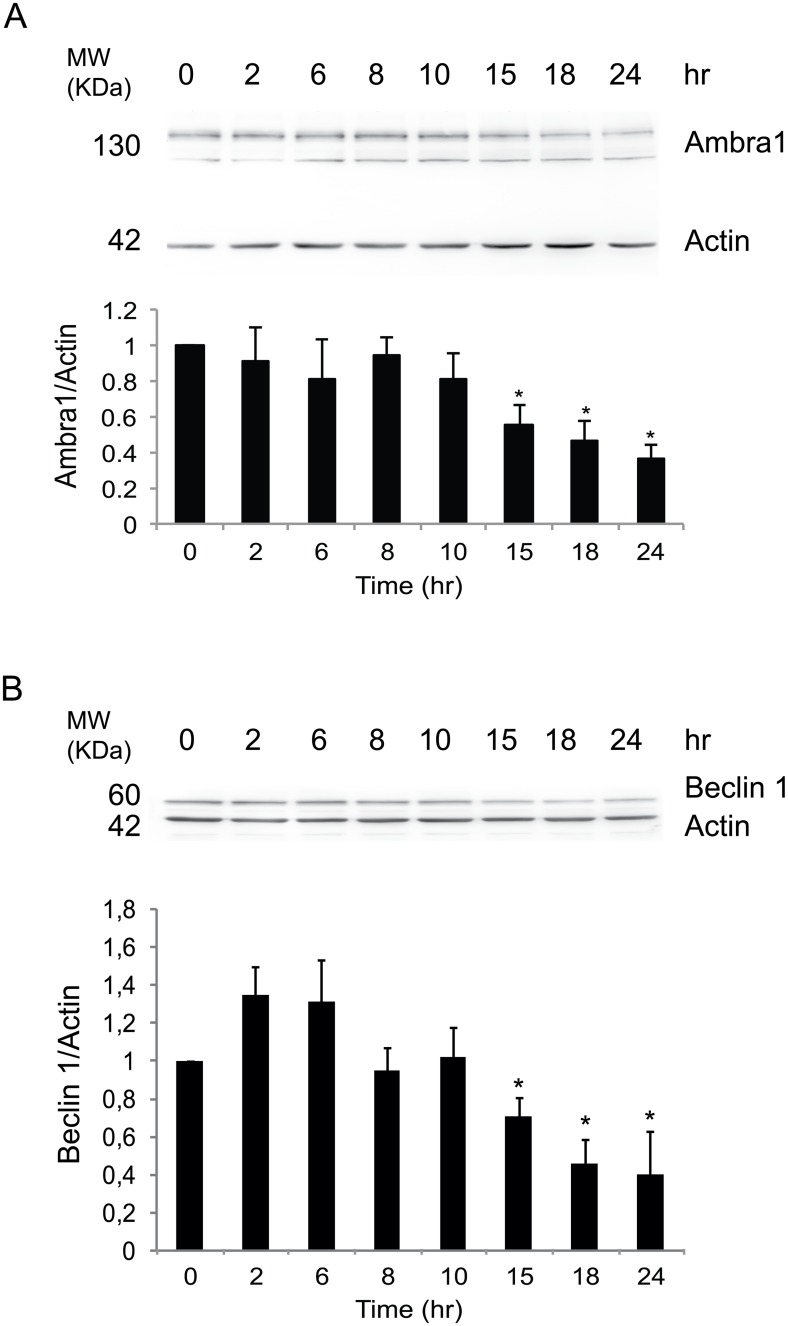
Persistent pseudohypoxia results in a gradual decrease of Ambra1 and Beclin 1 protein levels. HeLa cells were treated with 500 μM CoCl_2_ and Ambra1 and Beclin 1 protein were detected at 0, 2, 6, 8, 10, 15, 18, and 24 hr after treatment. The graph shows the densitometric quantification of Ambra1 (A) and Beclin 1 (B) related to Actin. Values are mean ± SD of three independent experiments relative to control. *: P-value < 0.05 is considered statistically significant.

**Fig 6:**
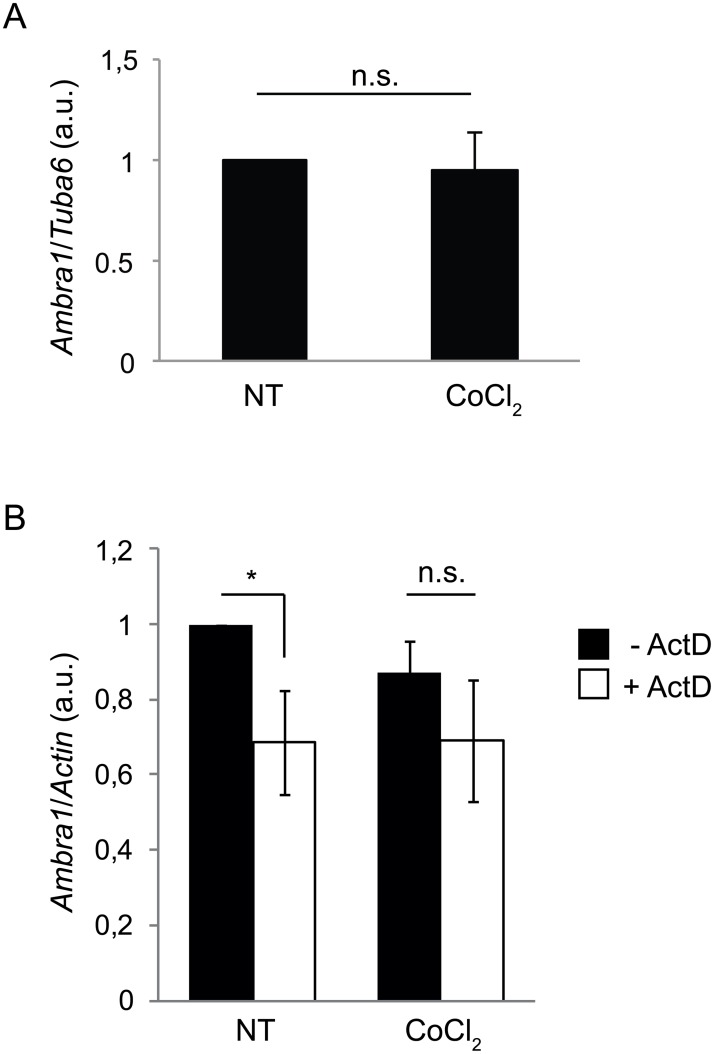
Ambra1 mRNA is stabilized after CoCl_2_ treatment. *Ambra1* mRNA level was analyzed by quantitative RT-PCR after 18 hr treatment. *Tuba6* mRNA was used for normalization (A). *Ambra1* mRNA level was analyzed by quantitative RT-PCR after adding Actinomycin D (ActD) for the last 4 hours of CoCl_2_ treatment; CoCl_2_ was left for 8 hours total at a concentration of 500 μM. *Actin* mRNA was used for normalization (B). a.u. = arbitrary units. Values are mean ± SD of three independent experiments relative to control. *: P-value < 0.05 is considered statistically significant, n.s. = not significant.

To investigate the regulation of autophagy genes upon hypoxia, the protein levels of Ambra1 and Beclin 1, two upstream proautophagic proteins, were analyzed by western blotting (WB; Fig 5A and 5B). A gradual decrease of Ambra1 and Beclin 1 protein levels was observed upon CoCl_2_ treatment. This finding is inversely related with what shown in Fig 2A, where we show an increase of LC3II protein levels starting at the 2h time-point and a continued increase until the final measurement at 24h. Next, we analyzed the *Ambra1* mRNA levels to verify whether these changes were due to transcriptional or translational regulation. Quantitative real-time PCR was performed using primers against *Ambra1* and *Tubulin α-6 (Tuba6)* (as a control). Interestingly, no significant changes in *Ambra1* mRNA levels were observed at 18h, the time-point at which protein levels decreased (Fig 6A). In order to understand if Ambra1 mRNA was somehow stabilized or instead transcribed and then degraded in a continuous loop, we performed an experiment using Actinomycin D (ActD), a drug inhibiting mRNA transcription. In the control sample, a treatment of 4 hours of ActD is sufficient to observe a significant decrease of *Ambra1* mRNA; However, on cells treated for 8 hours with CoCl_2_, ActD does not lead to a significant *Ambra1* mRNA decrease, this indicating that upon CoCl_2_ treatment there is a block of *Ambra1* mRNA production and degradation (Fig 6B).

**Fig 7:**
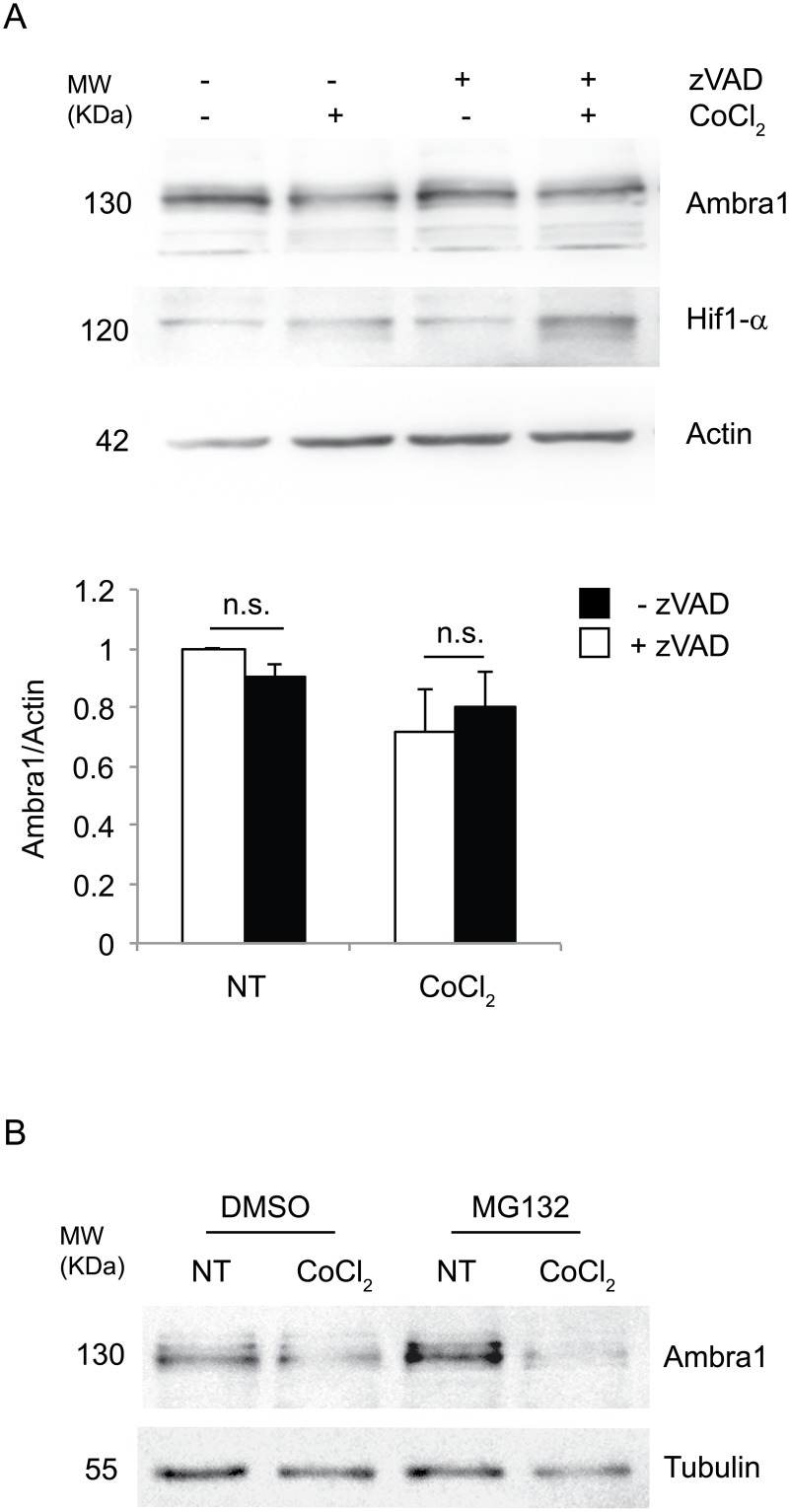
Inhibition of caspases or proteasome does not restore Ambra1 protein levels. HeLa cells were treated for 18h with 500 μM CoCl_2_ in the presence or absence of z-VAD-FMK (10 μM) (A) or MG132 (B). Induction of pseudohypoxia was confirmed by observing an increase in Hif1-α protein levels. The graph represents the densitometric analysis of Ambra1/Actin ratio in control and pseudohypoxic conditions with or without z-VAD-FMK (zVAD). Values are mean ± SD of three independent experiments relative to control. *: P-value < 0.05 is considered statistically significant (A). Experiments with MG132 were repeated three times with similar results (B).

A recent study has shown that long-term stress, such as starvation or staurosporin treatment, leads to Ambra1 protein degradation through caspase cleavage, this inhibiting the pro-survival role of autophagy [19]. To exclude the involvement of this event on the decrease of Ambra1 protein during prolonged pseudohypoxia, cells were treated with the pan-caspase inhibitor z-VAD-FMK along with CoCl_2_ for 18h. Interestingly, as shown in Fig 7A, inhibition of caspases had little if any effects on Ambra1 protein levels, implicating that other mechanism(s) must be responsible for Ambra1 protein decrease. Thus, we checked if the proteasome could account for Ambra1 decrease, by adding to the cells the proteasome inhibitor MG132 together with CoCl_2_; after 18 hours we observed that Ambra1 protein levels are still low when the proteasome is inhibited, this suggesting that the proteasome system is not responsible for AMBRA1 decrease (Fig 7B).

**Fig 8:**
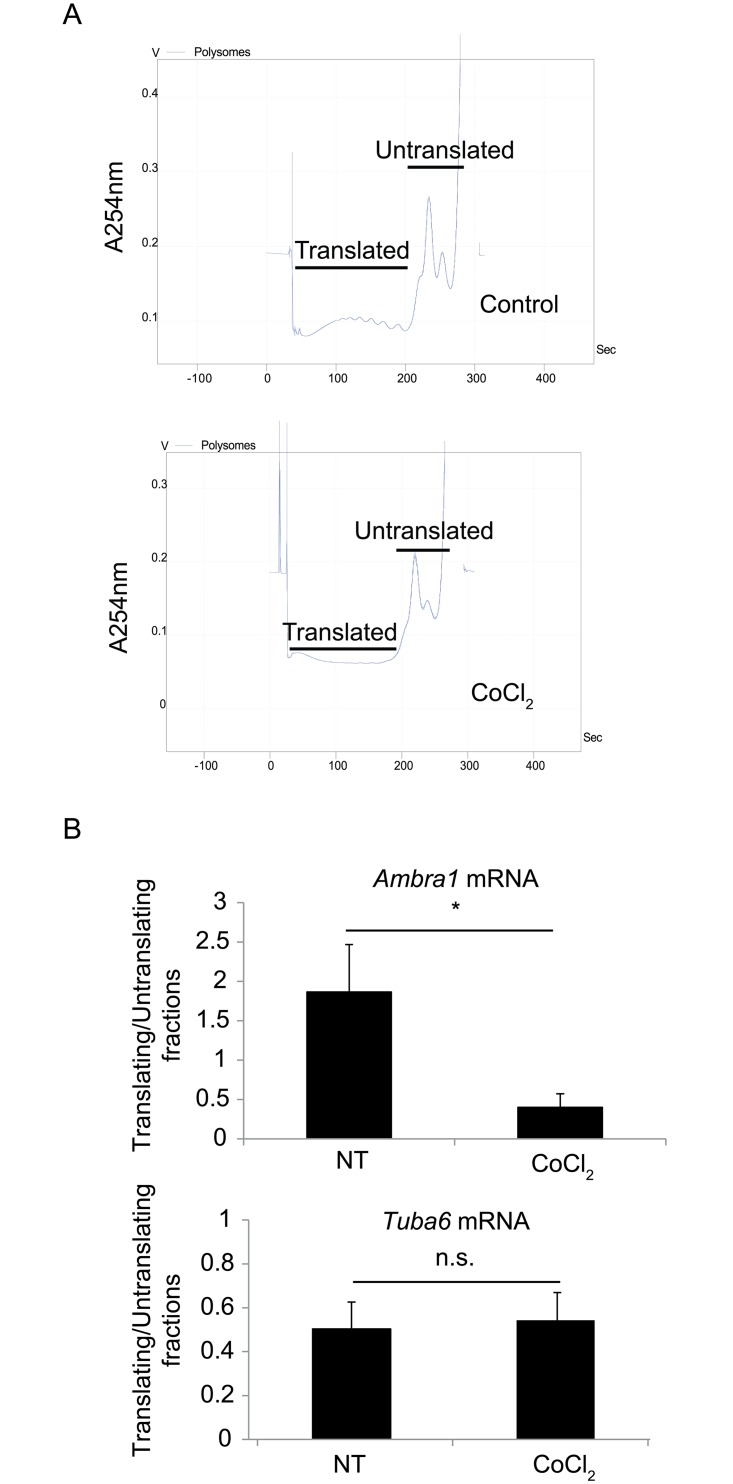
Accumulation of untranslated *Ambra1* mRNA after hypoxia. Polysome profiling was performed using control and hypoxic cells. Ten fractions were obtained from each sample by continuous OD_254_nm measurement (A). Quantitative RT-PCR was performed using mRNA extracted from translating (Translated) and non-translating (Untranslated) fractions (5 fractions each) using specific primers for *Ambra1* (B). Data were normalized with RNA-Spike. Values obtained from three independent experiments ± SD. *: Statistical significance. P-value < 0.05.

In principle, an apparent discrepancy between protein and mRNA levels implies the involvement of a translational control in this phenomenon. Therefore, we investigated the localization of *Ambra1* mRNA in normal and stress conditions. To check whether the transcription/translation rate of *Ambra1* mRNA was impacted by its translational control, *Ambra1* polysome profile was analyzed in both control conditions and 18h after induction of pseudohypoxia in HeLa cells. Ten fractions were obtained from each sample with continuous OD_254nm_ measurement. As shown in Fig 8A, pseudohypoxia resulted in a general decrease in translation. RNA was extracted from each fraction and the RNAs obtained from ‘translating’ fractions (#1–5) were pooled together. The same was done for ‘non-translating’ fractions (#6–10), followed by reverse transcription. Q-PCR showed a significant decrease in the amount of translating *Ambra1* mRNA after CoCl_2_ treatment, whilst control mRNA (*tuba6*) was unchanged in control and pseudohypoxic conditions (Fig 8B).

**Fig 9:**
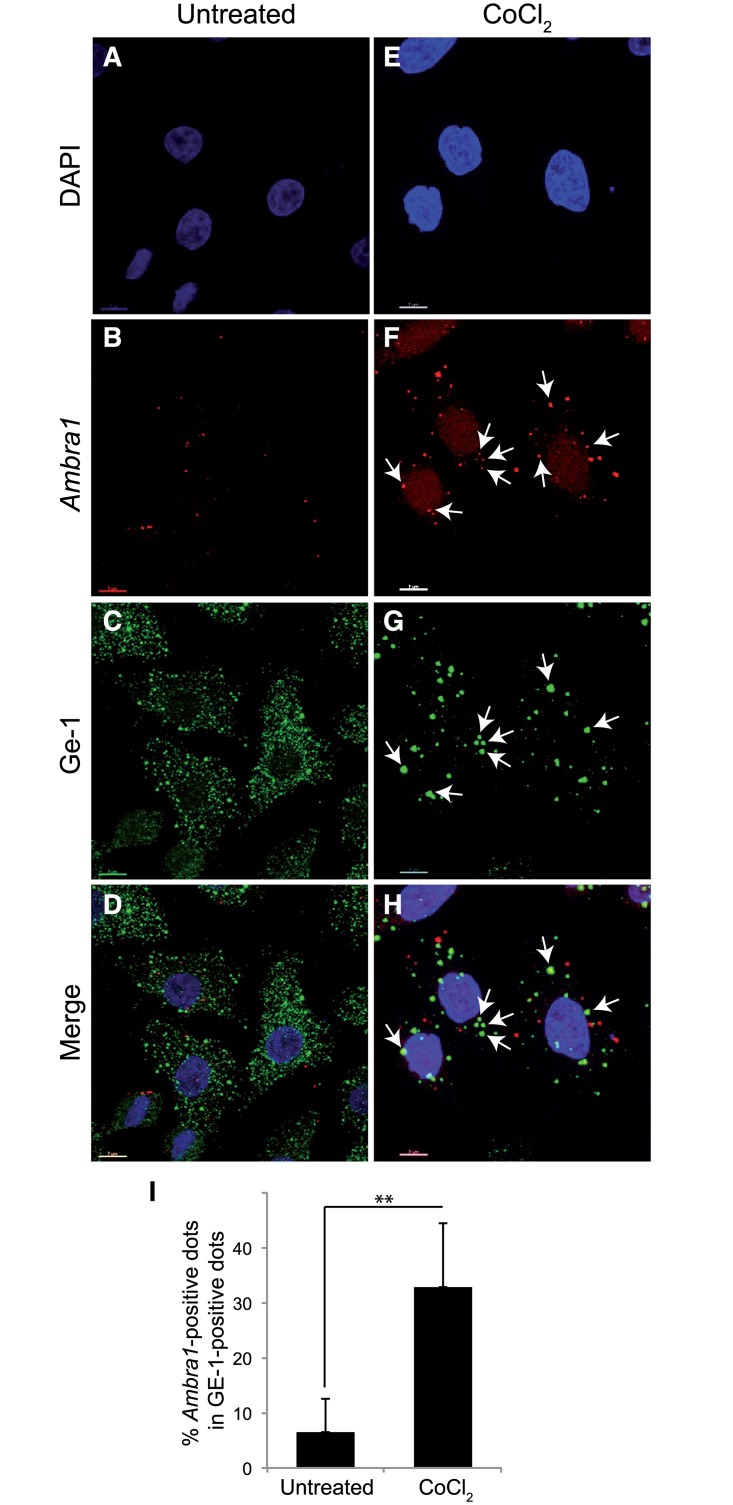
Translocation of *Ambra1* mRNA to cytoplasmic P-bodies upon pseudohypoxia. Left panels (A-D) represents control cells, whilst right panels (E-H) shows HeLa cells treated for 18 hr with 500 μM CoCl_2_. (C,G) Ge-1 protein; (B, F) *Ambra1* mRNA; (D, H) Merge image. Arrows show P-bodies (G), *Ambra1* mRNA (F), and co-localization of *Ambra1* and Ge-1 (H). The corresponding quantification in both conditions is shown in the graph below the images (I). **: Statistical significance. P-value < 0.005. Scale bars: 7 μm.

Non-translating mRNAs are often stored in cytoplasmic P-bodies. P-bodies are cytoplasmic structures that are involved in storage and processing of mRNAs [20]. They also play a role in translational regulation in different conditions [3]. We thus analyzed the localization of *Ambra1* mRNA in the cytoplasm before and after exposure to pseudohypoxic conditions (Fig 9). P-bodies were detected using an antibody against Ge-1 (a marker for this compartment) and appeared as fewer but much larger spots after CoCl_2_ treatment (Fig 9C and 9G). To analyze the localization of *Ambra1* mRNA relative to P-bodies, RNA-FISH was followed by immunostaining of Ge-1 (Fig 9B and 9F). Interestingly, while there is no co-localization at all between *Ambra1* mRNA and P-bodies in control conditions, pseudohypoxia induced by CoCl_2_ resulted in a partial re-localization of *Ambra1* mRNA to these cytoplasmic structures (see arrows in Fig 9D and 9H, Figure B in S2 Fig, and quantification of co-localization in both conditions in Fig 9I and Figure B in S2B Fig). As a control we performed the same analysis on *Beclin 1* mRNA, but, by contrast, no co-localization at all with P-bodies was observed (S3 Fig).

## Discussion

Here we propose the existence of a translational regulation of Ambra1, a pro-autophagy molecule, upon pseudohypoxia induction. We observed that Ambra1 protein levels decreased upon prolonged exposure to the hypoxia mimetic CoCl_2_, without any significant changes in mRNA levels. Along with data previously reported, we observed induction of pseudohypoxia in our cell system upon treatment with CoCl_2_, as confirmed by stabilization of Hif1-α protein.

Although it is widely accepted that Hif1-α activation leads to autophagy induction, after 8 hours of treatment with CoCl_2_ we found that LC3II accumulation is due to a block rather than to an induction of the autophagy flux, as revealed using chloroquine in western blot and in immunofluorescence experiments. This can be explained by the fact that, after a stress, autophagy is increased in order to preserve the cell, even though-if the stimulus persists for a long period, autophagy is blocked. Therefore, we can speculate that after 8 hours of pseudohypoxia, autophagy inhibition is due to the long exposure to CoCl_2_. On the contrary, p62 is slightly decreased after treatment, even if an increase in its transcription is observed, this being due, most likely, to hypoxia-mediated Nrf2 activation. This suggests a block in its translation or its protein degradation by caspases or the proteasome; alternatively, when autophagy is inhibited, p62 can forms aggregates that are insoluble in lysis buffer containing Triton. In addition, we show here that ROS are activated only after 16 hours of CoCl_2_ treatment, indicating that probably they are not early induced by CoCl_2_. Most likely, since an impaired degradation of damaged mitocondria leads to ROS production, we can speculate that the block of autophagy due to prolonged pseudohypoxia causes indirect ROS induction that, in turn, leads to apoptosis at later time points.

Looking at autophagy genes, we detected a gradual decrease in Ambra1 and Beclin 1 protein levels. We then proceeded with the analysis of Ambra1, a more upstream regulator of autophagy. Since *Ambra1* mRNA levels remain unchanged, the first hypothesis to test was degradation of the Ambra1 protein. A recent study by Piacentini and colleagues demonstrated that treating different cell lines such as *HeLa* and *2FGTH* with Staurosporine, a DNA-damage inducer, resulted in a gradual decrease of Ambra1 protein over time, without affecting the mRNA levels [19]. Interestingly, this decrease happened concomitantly with an increase in the PARP cleaved form, a marker of apoptosis induction. They further observed that Ambra1 was a target of caspases, since treating the cells with the caspase inhibitor z-VAD-FMK together with staurosporine could partially restore the level of Ambra1. Ambra1 degradation occurs when the stress is too intense or prolonged and autophagy cannot rescue the cell; therefore, by degradation of Ambra1, the pro-survival machinery of autophagy switches to apoptosis [19]. Although the direct interaction of Ambra1 with caspases has been reported, and inhibition of caspases prevents Ambra1 degradation in overexpressing conditions, it does not completely restore the endogenous protein levels [19]. This suggests the involvement of other mechanisms in down-regulation of Ambra1.

In this study, we have observed that long-term exposure to pseudohypoxic conditions results in the activation of apoptosis along with decreased Ambra1 protein levels, whilst mRNA level remains unchanged. Also, applying caspase inhibitor to the cells does not restore Ambra1 protein levels. We also showed that Ambra1 degradation is not due to the ubiqutine-proteasome system; in addition, we showed that MG132 together with CoCl_2_ leads to an almost complete depletion of Ambra1, suggesting that probably a protein involved in Ambra1 decrease is stabilized by blocking the proteasome-mediated degradation. Moreover, our experiments with ActD indicate that *Ambra1* mRNA transcription and turnover are blocked after the treatment with CoCl_2_.

Our data implies the involvement of a translational control in this phenomenon. To test this hypothesis, polysome profile analysis was performed and we observed translocation of *Ambra1* mRNA from polysomes (translating fraction) to non-translating fraction upon pseudohypoxia, resulting in the decrease of Ambra1 protein levels. Since un-translating mRNAs are often stored in cytoplasmic P-bodies, we analyzed the localization of *Ambra1* mRNA in the cytoplasm before and after exposure to hypoxic conditions. Even though *Ambra1* mRNA is stable in stress conditions, our data suggest that, at least in part, it is sequestered and stabilized in P-bodies, thus becoming inaccessible to the protein translation machinery. This sequestration may then result in a decrease in protein levels, in time with activation of cell death and suppression of the pro-survival pathway of autophagy. By contrast, *Beclin 1* mRNA does not relocate to P-bodies at all in similar conditions, indicating that the mechanism here described does not concern all autophagy regulators. Although in general the decay of mRNAs is a consequence of prolonged stress conditions that lead to apoptosis, it is plausible that the maintenance of a pool of *Ambra1* mRNA may represent an important reserve for the cell in case normal conditions are restored. Indeed, this would allow *Ambra1* prompt translation.

As for the mechanism by which *Ambra1* mRNA moves to P-bodies, it certainly needs further investigation. There is evidence for interaction of P-bodies with actin filaments and/or microtubule structures [21,22]. Also, association of certain RNAs with the dynein motor complex for dendritic localization has been postulated [23]. Therefore, the next step would be studying the effect of microtubule disruption on localization of *Ambra1* mRNA with or without CoCl_2_ treatment and its effect on cell death and survival in hypoxic conditions. Furthermore, since the co-localization of *Ambra1* mRNA and P-bodies after CoCl_2_ treatment is only partial, it would be interesting to know if this messenger can also localize in other specific foci, such as stress granules.

## Supporting Information

S1 Fig Autophagy flux is blocked after long exposure to CoCl_2_. Representative images of HeLa cells not treated (NT) ore treated with CoCl_2_ 500 μM for 8 hours in presence or not of chloroquine 20 μM (CHQ) for the last hour of treatment.
(EPS)

S2 Fig Detailed analysis of *Ambra1* mRNA into cytoplasmic P-bodies after hypoxia. (A) The image represents 3D-analysis of *Ambra1* mRNA (red) and Ge-1 protein (green) co-localization after 18 hr treatment with CoCl_2_. Blue: Nuclear staining with DAPI. (B) total *Ambra1*-positive dots were counted in treated and untreated cells: the graph shows that their total number is unchanged.
(EPS)

S3 Fig Upon CoCl_2_ treatment Beclin 1 mRNA does not traslocate to P-bodies. Left panels (A-C) represents control cells, whilst right panels (D-F) shows HeLa cells treated for 18 hr with 500 μM CoCl_2_. (B, E) Ge-1 protein; (A, D) *Beclin 1* mRNA; (C, F) Merge image. Scale bars: 10 μm
(EPS)

## References

[1] Giaccia AJ, Simon MC, Johnson R (2004) The biology of hypoxia: the role of oxygen sensing in development, normal function, and disease. Genes Dev 18: 2183–2194. 15371333 10.1101/gad.1243304 PMC517513

[2] Semenza GL (2012) Hypoxia-inducible factors in physiology and medicine. Cell 148: 399–408. 10.1016/j.cell.2012.01.021 22304911 PMC3437543

[3] Bellot G, Garcia-Medina R, Gounon P, Chiche J, Roux D, Pouysségur J, et al (2009) Hypoxia-induced autophagy is mediated through hypoxia-inducible factor induction of BNIP3 and BNIP3L via their BH3 domains. Mol Cell Biol 29: 2570–2581. 10.1128/MCB.00166-09 19273585 PMC2682037

[4] Forsythe JA, Jiang BH, Iyer NV, Agani F, Leung SW, Koos RD, et al (1996) Activation of vascular endothelial growth factor gene transcription by hypoxia-inducible factor 1. Mol Cell Biol 16: 4604–4613. 8756616 10.1128/mcb.16.9.4604 PMC231459

[5] Semenza GL, Wang GL (1992) A nuclear factor induced by hypoxia via de novo protein synthesis binds to the human erythropoietin gene enhancer at a site required for transcriptional activation. Mol Cell Biol 12: 5447–5454. 1448077 10.1128/mcb.12.12.5447-5454.1992 PMC360482

[6] Esclatine A, Chaumorcel M, Codogno P (2009) Macroautophagy signaling and regulation. Curr Top Microbiol Immunol 335: 33–70. 10.1007/978-3-642-00302-8_2 19802559

[7] Fimia GM, Stoykova A, Romagnoli A, Giunta L, Di Bartolomeo S, Nardacci R, et al (2007) Ambra1 regulates autophagy and development of the nervous system. Nature 447: 1121–1125. 17589504 10.1038/nature05925

[8] Strappazzon F, Vietri-Rudan M, Campello S, Nazio F, Florenzano F, Fimia GM, et al (2011) Mitochondrial BCL-2 inhibits AMBRA1-induced autophagy. EMBO J 30: 1195–1208. 10.1038/emboj.2011.49 21358617 PMC3094111

[9] Di Bartolomeo S, Corazzari M, Nazio F, Oliverio S, Lisi G, Antonioli M, et al (2010) The dynamic interaction of AMBRA1 with the dynein motor complex regulates mammalian autophagy. J Cell Biol 191(1): 155–68. 10.1083/jcb.201002100 20921139 PMC2953445

[10] He C, Klionsky DJ (2009) Regulation mechanisms and signaling pathways of autophagy. Annu Rev Genet 43: 67–93. 10.1146/annurev-genet-102808-114910 19653858 PMC2831538

[11] Carbonaro M, O’Brate A, Giannakakou P (2011) Microtubule disruption targets HIF-1alpha mRNA to cytoplasmic P-bodies for translational repression. J Cell Biol 192: 83–99. 10.1083/jcb.201004145 21220510 PMC3019555

[12] Valencia-Sanchez MA, Liu J, Hannon GJ, Parker R (2006) Control of translation and mRNA degradation by miRNAs and siRNAs. Genes Dev 20: 515–524. 16510870 10.1101/gad.1399806

[13] Holt CE, Bullock SL (2009) Subcellular mRNA Localization in Animal Cells and Why it Matters. Science 326(5957): 1212–6. 10.1126/science.1176488 19965463 PMC3785123

[14] Czaplinski K, Singer RH (2006) Pathways for mRNA localization in the cytoplasm. Trends Biochem Sci 31: 687–693. 17084632 10.1016/j.tibs.2006.10.007

[15] Brengues M, Teixeira D, Parker R (2005) Movement of eukaryotic mRNAs between polysomes and cytoplasmic processing bodies. Science 310: 486–489. 16141371 10.1126/science.1115791 PMC1863069

[16] Parker R, Sheth U (2007) P Bodies and the Control of mRNA Translation and Degradation. Mol Cell 25: 635–646. 17349952 10.1016/j.molcel.2007.02.011

[17] Battaglia V, Compagnone A, Bandino A, Bragadin M, Rossi CA, Zanetti F, et al (2009) Cobalt induces oxidative stress in isolated liver mitochondria responsible for permeability transition and intrinsic apoptosis in hepatocyte primary cultures. Int J Biochem Cell Biol 41: 586–594. 10.1016/j.biocel.2008.07.012 18708157

[18] Naves T, Jawhari S, Jauberteau M, Verdier M (2013) Autophagy takes place in mutated p53 neuroblastoma cells in response to hypoxia mimetic CoCl 2. Biochem Pharmacol 85: 1153–1161. 10.1016/j.bcp.2013.01.022 23380477

[19] Pagliarini V, Wirawan E, Romagnoli A, Ciccosanti F, Lisi G, Lippens S, et al (2012) Proteolysis of Ambra1 during apoptosis has a role in the inhibition of the autophagic pro-survival response. Cell Death Differ 19: 1495–1504. 10.1038/cdd.2012.27 22441670 PMC3422474

[20] Balagopal V, Parker R (2009) Polysomes, P bodies and stress granules: states and fates of eukaryotic mRNAs. Curr Opin Cell Biol 21: 403–408. 10.1016/j.ceb.2009.03.005 19394210 PMC2740377

[21] Loschi M, Leishman CC, Berardone N, Boccaccio GL (2009) Dynein and kinesin regulate stress-granule and P-body dynamics. J Cell Sci 122: 3973–3982. 10.1242/jcs.051383 19825938 PMC2773196

[22] Kanai Y, Dohmae N, Hirokawa N (2004) Kinesin transports RNA: Isolation and characterization of an RNA-transporting granule. Neuron 43: 513–525. 15312650 10.1016/j.neuron.2004.07.022

[23] Xu X, Brechbiel JL, Gavis ER (2013) Dynein-dependent transport of nanos RNA in Drosophila sensory neurons requires Rumpelstiltskin and the germ plasm organizer Oskar. J Neurosci 33: 14791–14800. 10.1523/JNEUROSCI.5864-12.2013 24027279 PMC3771026

